# A Prospective, Randomized, Multicenter Trial Assessing a Novel Lysine-Derived Urethane Adhesive in a Large Flap Surgical Procedure without Drains

**DOI:** 10.1007/s00266-015-0498-4

**Published:** 2015-06-05

**Authors:** Joseph P. Hunstad, Joseph Michaels, A. Jay Burns, Sheri Slezak, W. Grant Stevens, Dottie M. Clower, J. Peter Rubin

**Affiliations:** Department of Surgery, Division of Plastic Surgery, and the Hunstad Kortesis Center for Cosmetic Plastic Surgery and Med Spa, The University of North Carolina Chapel Hill, Huntersville, NC USA; Michaels Aesthetic & Reconstructive Plastic Surgery, Bethesda, MD USA; Dallas Plastic Surgery Institute, Dallas, TX USA; Division of Plastic Surgery, University of Maryland School of Medicine, Baltimore, MD USA; Marina Plastic Surgery Associates, Marina Del Ray, CA USA; Cohera Medical, Pittsbugh, PA USA; Department of Plastic Surgery, University of Pittsburgh School of Medicine, 690 Scaife Hall, 3550 Terrace Street, Pittsburgh, PA 15261 USA

**Keywords:** Flap surgery, Abdominoplasty, Drains, Adhesive, TissuGlu, Seroma

## Abstract

**Objective:**

To evaluate the safety and effectiveness of a lysine-derived urethane adhesive as a noninvasive alternative to closed suction drains in a commonly performed large flap surgical procedure.

**Methods:**

One hundred thirty subjects undergoing abdominoplasty at five centers were prospectively randomized to standard flap closure with surgical drains (Control group) or a lysine-derived urethane adhesive (Treatment group) without drains. The primary outcome measured was the number of post-operative procedures, including drain removals (as the event marking the use of a surgical drain) and needle aspirations. Secondary endpoints included total wound drainage, cumulative days of treatment, and days to drain removal. A patient questionnaire evaluating quality of life measures was also administered.

**Results:**

Subjects in the Treatment group required significantly fewer post-operative procedures compared to the Control group (1.8 ± 3.8 vs. 2.4 ± 1.2 procedures; *p* < 0.0001) and fewer cumulative days of treatment (1.6 ± 0.4 vs. 7.3 ± 3.3; *p* < 0.0001). A procedure to address fluid accumulation was required for only 27.3 % of the subjects in the Treatment group versus 100 % of Control group, which by study design required the use of drains. The mean duration of use of indwelling surgical drains for the Control group was 6.9 ± 3.3 days. All fluid collections treated with percutaneous aspiration were resolved and there were no unanticipated adverse events.

**Conclusion:**

The results of the study support that the use of a lysine-derived urethane adhesive is a safe and effective alternative to drains in patients undergoing a common large flap surgical procedure.

**Level of Evidence I:**

This journal requires that authors assign a level of evidence to each article. For a full description of these Evidence-Based Medicine ratings, please refer to the Table of Contents or the online Instructions to Authors www.springer.com/00266.

## Introduction

Many surgical procedures involve the creation of large tissue flaps. One of the drawbacks of these large tissue flaps is that they have a high incidence of fluid accumulation-related complications [1–5]. The accumulation of serous fluid in the space between the elevated tissue flap and the underlying tissue (the “dead space”) can physically prevent apposition between these two tissue layers and interfere with healing. This results in an increased risk of early post-operative complications including seroma formation, necrosis, and wound breakdown [6–8]. These complications may require multiple interventions, including repeated needle aspirations, or in some cases may require surgical intervention to eradicate the dead space and enable wound healing. The most common of these complications, seroma formation, can occur in 15–52 % of the patients following commonly performed large flap procedures [1–5].

The standard of care for managing wound drainage and preventing fluid accumulation following large flap procedures is the placement of surgical drains to provide continuous egress of fluid from the dead space during the immediate post-operative period [9–11]. Drains are typically left in place for a specific duration of time following surgery or until fluid output drops below a specified level. While drains are effective at removing fluid, they do not seal or eliminate the dead space between tissue layers following large flap procedures, or prevent shearing forces from disrupting the early healing process during movement. As a result, up to 52 % of the patients with drains will still require invasive procedures in order to manage post-operative fluid complications [9–11]. Importantly, the use of drains is associated with a significant increase in the post-operative pain and hospital stays while also increasing the risk for retrograde bacterial migration and infection [12–14]. Perhaps the most serious of these risks is surgical site infection associated with prolonged drain use [15–17]. The use of drains can lead to the development of increased post-operative pain and discomfort, as well as additional scarring at the drain insertion site. Indwelling drains may also impact quality of life, interfere with sleep, and delay the return to normal activities [14].

A novel lysine-derived urethane adhesive (TissuGlu^®^ Surgical Adhesive, Cohera Medical, Inc., Pittsburgh, PA) has recently been developed which adheres large tissue flaps to the underlying tissue during surgical procedures [18–20]. This biocompatible adhesive provides tight bonding of tissue to reduce the dead space between tissue layers and minimizes shearing forces during the healing process. The adhesive is non-toxic and has a bonding strength equivalent to cyanoacrylates. The use of the adhesive may provide an alternative to closed suction drains, reducing patient discomfort and the risks associated with indwelling drains. This prospective, randomized clinical trial compares the outcomes in patients undergoing large flap surgery using closed suction drains to patients undergoing large tissue flap surgery using the lysine-derived urethane adhesive without drains.

Elective abdominoplasty was selected as the representative large flap surgical procedure for this study since it allowed for a homogenous study population, reducing the likelihood of confounding variables which would impact the measured outcomes. Specifically, elective abdominoplasty is typically performed on healthy patients using standardized surgical technique with the creation of a large fasciocutaneous flap with a similar amount of dead space among patients. In addition to elective abdominoplasty being a very reproducible procedure, the tissue flap elevated is larger than that seen in most other large flap procedures, and because the flap is approximated with the patient in a flexed position, it involves significant shearing forces. Additionally, this is a procedure for which post-operative fluid accumulation is a known risk, with numerous publications describing the rates of occurrence.

## Methods

### Study Design

Following Investigational Review Board approval and registration of the study with the National Institutes of Health’s clinical trial registry (NCT01526954), 130 subjects undergoing elective abdominoplasty at five centers in the United States were enrolled in the study. All study subjects provided written informed consent for the participation in the study prior to enrollment. Following receipt of informed consent, subjects were randomized at each center to receive either standard wound closure with drains (Control group) or a lysine-derived urethane adhesive (Treatment group) without drains while undergoing the abdominoplasty procedure. Randomization was performed pre-operatively using randomization envelopes and subjects were not informed of their randomization assignment prior to surgery. Since the study protocol randomized subjects to the use of drains versus a therapy without drains, it was not possible for subjects or investigators to be blinded to the treatment groups.

### Study Population

All subjects were required to be 18 years of age or older, in good general health with no conditions that would significantly impact wound healing, have a body mass index (BMI) ≤28, and were scheduled for at least one full thickness surgical incision of at least 20 cm in length as part of an elective standard abdominoplasty procedure. Subjects were excluded from the study if they were a current smoker, had undergone previous abdominoplasty, prior bariatric or weight loss surgery, or had lost ≥15 % of maximum lifetime bodyweight (excluding pregnancy weight gain). Additional exclusion criteria included serious comorbid conditions such as heart disease, blood clotting disorder, or diabetes.

### Surgical Technique

The surgical procedure was consistent with the standard techniques used for an abdominoplasty procedure [21]. For the Control group, two 15 French Blake^®^ drains (Ethicon, Inc., New Brunswick, New Jersey, USA) were placed over the abdominal fascia for all subjects and the tube was delivered through stab incisions on the pubic area. For the Treatment group, the lysine-derived urethane adhesive was applied in a grid-like pattern to the exposed abdominal fascial surface immediately prior to closing the flap as per the manufacturer’s instructions, avoiding the area under the incision [22]. The abdominal flap was then approximated over the abdominal tissue surface and temporarily secured at the incision line to prevent movement while the low transverse incision was closed, ensuring minimal pressure or disruption of the flap during the remainder of the procedure in order to prevent smearing of the adhesive across the entire tissue surface. No quilting sutures or fibrin sealants were used for subjects enrolled in either arm of the study. All subjects received similar post-operative care.

### Outcome Measures

The present study was designed as a non-inferiority study in order to test the hypothesis that the use of the lysine-derived urethane adhesive, without the concomitant use of drains, could provide a less invasive alternative to the use of drains for the management of fluid accumulation following a common large flap surgical procedure. In order to normalize the treatment modalities and effectively compare the two groups, the primary endpoint for the study was defined as the number of post-operative invasive procedures. Removal of an indwelling drain was counted as a single invasive procedure, as was a percutaneous needle aspiration. This design takes into account the fact that an indwelling drain is an invasive method of managing post-operative fluid. While it would be possible to design an outcome measure that takes into account the number of days an indwelling drain was in place, this study design focused on singular events that could be reasonably compared. As a result, removal of one drain was scored as a single invasive procedure regardless of the number of days it had been in place and represents the entire treatment process of having that drain in place.

Using the number of post-operative invasive procedures, the frequency of invasive management of the fluid-related complications (such as needle aspiration or removal of post-operative drains) can be captured and compared in an unbiased manner between test and control conditions. Our study hypothesis was that the elimination of dead space in the wound in the Treatment group would reduce the number of invasive procedures related to post-operative fluid management and the Treatment group would not be inferior to the Control group.

Secondary endpoints for the study included cumulative drain volume, aspiration volume, total wound drainage (drain volume + aspiration volume), cumulative days of invasive treatment (days with drains in + days aspirated), and days to drain removal. A patient questionnaire that evaluated Quality of Life measures related to resuming normal activities was also administered during the study. Seroma rate (defined as a clinically detectable palpable fluid collection requiring needle aspiration) was also documented. However, it should be recognized that in a treatment paradigm that involves an adhesive and not drains, percutaneous aspiration is the only method used for managing post-operative fluid collections, and that any fluid collection treated is classified as a seroma. Conversely, in the Control group, all subjects have indwelling drains, an invasive treatment to control fluid, and seroma is only diagnosed and treated with a second invasive therapy after the drains are removed.

### Data Collection

Subjects were assessed by physical examination at 12 time points starting at post-operative day 3(±1) and ending on post-operative day 84 (±3), with the assessments being more frequent during the immediate post-operative period. In order to ensure that subjects with fluid-related complications were managed to current best practices, subjects with a drain in place or subjects that required an aspiration during an office visit were automatically scheduled for an additional follow-up visit 3 ± 1 days later. Once the drain was removed or the seroma resolved, the subject reverted to the regular follow-up schedule. A Quality of Life patient questionnaire was administered during each scheduled follow-up subject visit.

### Statistical Analysis

The primary and secondary endpoints, including the safety endpoints, were analyzed using the intent to treat (ITT) subject group which included all enrolled subjects regardless of their adherence to the follow-up regimen. The statistical analysis of the primary efficacy endpoint consisted of a between-group comparison of the number of invasive procedures to assess the non-inferiority of treatment to control subjects. Using non-parametric methods, the difference in median number of invasive procedures was compared to a non-inferiority margin of one. An exact two-sample Wilcoxon test was used to statistically assess the non-inferiority endpoint. Reported p-values are two-sided, and statistical significance was assessed at an alpha level of 0.05. In the case where the primary non-inferiority endpoint was met, an additional supportive analysis of superiority of the primary endpoint was performed.

Continuous data were summarized using descriptive statistics such as mean, standard deviation, median, and range. Counts and percentages were used to summarize categorical data. Statistical comparisons between the treatment groups were made using two-sample Wilcoxon tests (continuous data) or Fisher’s exact tests (categorical data).

## Results

### Subject Demographics

**Fig. 1:**
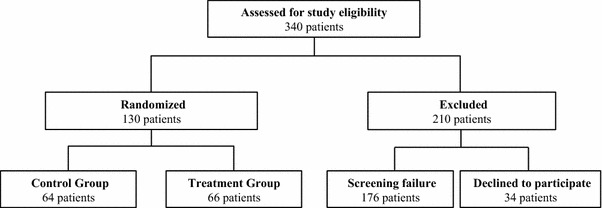
Flow of study participants

A total of 130 subjects were enrolled in the study (Fig. 1). There were no statistical differences between the Treatment and Control groups for any demographic, medical, or procedure-related factors. The mean age and BMI for the Treatment and Control groups were 42.1 ± 8.4 versus 42.6 ± 10.6 years (*p* = 0.961) and 24.2 ± 2.4 versus 24.5 ± 2.0 (*p* = 0.445), respectively (Table 1). The mean time to apply the lysine-derived urethane adhesive for subjects in the Treatment group was 1.2 ± 0.9 min. There was no significant difference in overall time to perform the abdominoplasty procedure between the Treatment and Control groups (102.3 ± 44.5 vs. 105.7 ± 47.7 respectively; *p* = 0.755) (Table 2).

**Table 1:** Baseline demographics

	Control group	Treatment group	*p* value
	(*n* = 64)	(*n* = 66)	
Age (years)	42.6 ± 10.6	42.1 ± 8.4	0.9610
Female (gender)	63/64 (98.4 %)	66/66 (100.0 %)	0.4923
Weight (kg)	65.4 ± 7.8	65.0 ± 7.6	0.9258
Height (cm)	163.2 ± 7.0	163.9 ± 5.9	0.3981
BMI	24.5 ± 2.0	24.2 ± 2.4	0.4453
Lifetime body weight loss (%)	4.2 ± 4.2	3.8 ± 5.0	0.2727
History of surgical procedures	53/64 (82.8 %)	53/66 (80.3 %)	0.8222

**Table 2:** Procedural Data

	Control group	Treatment group	*p* value
	(*n* = 64)	(*n* = 66)	
Procedure time (min)	105.7 ± 47.7	102.3 ± 44.5	0.7550
Incision length (cm)	41.2 ± 9.7	40.8 ± 9.1	0.9480
Abdominal flap thickness (mm)	26.0 ± 8.1	27.6 ± 8.1	0.2057
Weight of tissue removed (lb)	1.3 ± 0.8	1.4 ± 1.0	0.8304

### Clinical Outcomes—Primary Endpoint

**Fig. 2:**
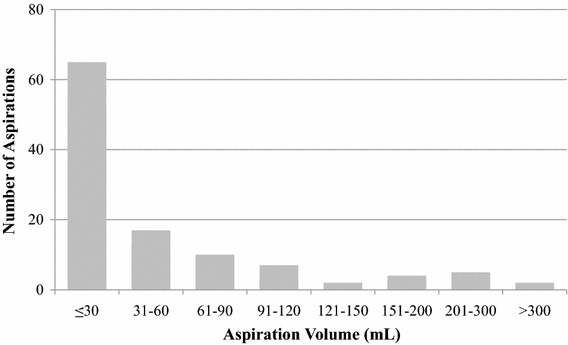
Distribution of needle aspirations in Treatment group by aspiration volume. 58 % of aspirations were for volumes ≤30 mL

**Fig. 3:**
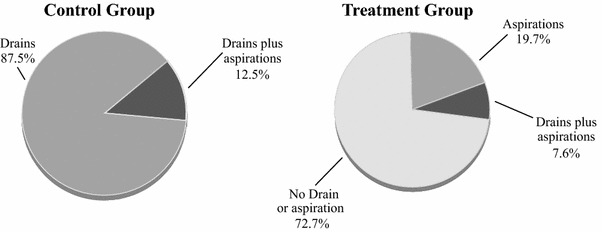
Comparison of procedures to manage fluid in the operative field

Table 3 reports the number of invasive procedures for subjects in the Control and Treatment groups. The Control group required 152 procedures including 128 drain removals and 24 aspirations. The Treatment group required 119 procedures including 7 drain removals and 112 aspirations. The number of invasive procedures in the Treatment group was found to be non-inferior (*p* < 0.0001) to the Control group. Given that the non-inferiority criterion was met, the pre-specified evaluation of superiority was also performed. Our data documented that the Treatment group was superior to the Control group (*p* < 0.0001) relative to the total number of post-operative procedures. Of note, 58 % of the aspirations in the Treatment group were for low fluid volumes, defined as less than 30 mL, the standard threshold considered to justify drain removal (Fig. 2). An invasive procedure to address fluid accumulation was required in only 27.3 % of the subjects in the Treatment group versus 100 % in the Control group (Fig. 3).

**Table 3:** Primary efficacy endpoints: number of invasive treatments

	Control group (*n* = 64)		Treatment group (*n* = 66)		Comparison
	Events per patient	Total # of events	Events per patient	Total # of events	*p* value^*^
Number of post-operative invasive procedures	2.4 ± 1.2 (2.0)	152	1.8 ± 3.8 (0.0)	119	<0.0001
Needle aspirations	0.4 ± 1.2 (0.0)	24	1.7 ± 3.7 (0.0)	112	NA
Drain removal	2.0 ± 0.0 (2.0)	128	0.1 ± 0.4 (0.0)	7	NA

Summary statistics are presented as Mean ± SD (Median)

* *p* values are from exact Wilcoxon test of non-inferiority as well as superiority comparing Treatment Group to Control group. Reported* p* values are 2-sided

### Clinical Outcomes—Secondary Endpoint

In Table 4, the results of the secondary endpoint analysis for the entire study population of 130 subjects are shown. Cumulative drainage volume (including both drain volume and aspiration volume) was significantly less in the Treatment group compared to the Control group (96.8 ± 270.1 vs. 411.4 ± 366.6; *p* < 0.0001). The cumulative number of invasive treatment days (days with drains in + days aspirated) was also significantly less in the Treatment group (1.6 ± 3.4 vs. 7.3 ± 3.3; *p* < 0.0001). Subjects in the Control group required indwelling surgical drains for 6.9 ± 3.3 days.

**Table 4:** Secondary endpoints: drainage volume and seroma management

	Control group	Treatment group	*p* value
	(*n* = 64)	(*n* = 66)	
Total wound drainage	411.4 ± 366.6	96.8 ± 270.1	<0.0001
Cumulative drain volume	396.5 ± 339.9	–	N/A
Aspiration volume	14.9 ± 67.1	96.8 ± 270.1	0.0202
Days to drain removal	6.9 ± 3.3	–	N/A
Cumulative days of invasive treatment	7.3 ± 3.3	1.6 ± 3.4	<0.0001

*p* values are from two-sample Wilcoxon test

As anticipated as a result of the study design, the rate of seroma formation (by classic definition, a palpable fluid collection) was statistically significantly higher in the Treatment group when compared to the Control group (27.3  vs. 12.5 %; *p* = 0.0479). Drains were placed in five subjects in the Treatment group. This included one subject who developed a hematoma unrelated to the lysine-derived urethane adhesive and two subjects where drains were placed after low-volume fluid accumulation (72 mL in one subject and 82 mL in the other). Drains were placed in the remaining two subjects in the Treatment group as a result of high volume fluid accumulation.

No subjects in either group developed a surgical site infection and only one subject (in the Treatment group) developed a post-operative wound infection. There were no statistical differences in the rate of wound dehiscence, surgical site infection, skin necrosis, hematoma formation, or wound complications between the two groups.

### Quality of Life (QOL) Questionnaires

QOL questionnaires were administered at each scheduled follow-up visit to assess whether the subject had resumed normal activities such as showering, driving, exercising, walking up stairs, or returning to work. While the current study was not powered to demonstrate statistical significances in QOL measures, the mean and median of time to the resumption of all measured activities was lower in the Treatment group compared to the Control group (data not shown).

## Discussion

The risk of fluid-related complications after large flap surgical procedures such as abdominoplasty is well documented, with incidences ranging from 15 to 52 % [1–5]. In this subset of patients, subcutaneous fluid continues to accumulate in the wound, leading to persistent seroma formation. The term seroma is inconsistently defined, but in general is considered to be a palpable collection of excess fluid between the tissue layers that cannot be fully absorbed [22, 23]. Seroma formation is regulated by the balance of secretion and reabsorption of fluid, and in cases where reabsorption cannot keep up with fluid output, the patient may require repeated aspirations to remove fluid from the wound. Excess fluid secretion leading to seroma formation is believed to result from the dead space created between the tissue layers during surgery, as well as from shearing forces between the underlying tissue and the abdominal flap after surgery [4, 24].

The results of this large randomized, prospective study support the use of the lysine-derived urethane adhesive as a safe and effective alternative to drains in subjects undergoing a common large flap surgical procedure. Subjects treated with the adhesive were able to achieve comparable outcomes compared to subjects with drains, while experiencing a decrease in the total number of invasive procedures required to manage postsurgical fluid accumulation-related issues. The cumulative number of days receiving treatment was also significantly less in the Treatment group since they did not require the placement of drains. In the Treatment group, 27 % of the subjects required invasive procedures, whereas in the Control group, 100 % required invasive procedures.

As is the convention in adverse event recording, aspiration was treated as a surrogate for seroma in our study, such that any time an aspiration was performed on a subject, the patient was counted as having a seroma. To avoid bias and insure consistency across study sites, a seroma was determined as present or absent independent of its volume, timing, or degree of risk to the subject. The higher seroma rate noted in the Treatment group was likely due to the difference in the way fluid removal was categorized between the two groups. Since the Treatment group had no drains, any fluid removal in this group (e.g. by aspiration) was categorized as a seroma, whereas removal of fluid by a drain in the Control group was considered as drainage volume only and not categorized as seroma. While drains were placed post-operatively for seroma formation in 4 subjects randomized to the Treatment arm, two of these subjects had very low total volumes of fluid aspirated (<100 mL cumulative volume) prior to drain placement with the drains likely placed due to an overabundance of caution associated with the initial use of the adhesive without drains. The other two subjects who had drains placed were from the same study site where a higher volume (up to 500 mL) of tumescent fluid was utilized during the procedure compared to other sites (<200 mL). This increased volume of tumescent fluid used may have contributed to the excess fluid accumulation observed in these two subjects.

Current approaches to reduce seroma risk have focused on eliminating fluid from the wound during the days after surgery. For decades, closed suction drainage has been considered the standard of care for the prevention of fluid-related complications [12, 24]. Surgical drains typically remain in place for a week or more until the volume of fluid collected drops below a specific level. This amount is generally established as less than 30 ml of fluid measured in a 24-h period [25, 26]. Comparing five studies in which the mean time to last drain removal after abdominoplasty was measured, the average time to last drain removal per subject was 6.9 days [4, 27–30].

Although well accepted as the standard of care, the use of closed suction drains is not without risks. The use of drains is associated with a significant increase both in post-operative pain and hospital stay, as well as complications including retrograde bacterial migration and infection [13, 14]. Perhaps the most serious of these is the increased risk of surgical site infections (SSIs) that is associated with drain use [15–17]. In a study of breast biopsy patients, the use of post-operative drains was one of the risk factors most highly associated with SSIs, and 26 % of the patients developing SSIs required re-hospitalization for treatment [31]. Studies indicate a correlation between the length of time the drain remains in place and the likelihood of developing a SSI [32, 33]. For example, in a retrospective study of breast surgeries, drains remaining in place for 5–15 days increased the odds ratio of developing a SSI to 1.84 compared to patients with no drains, with a further increase to an odds ratio of 2.14 for drains in longer than 16 days [33].

An alternative, but less accepted method which has been explored for reducing dead space and the likelihood of seroma formation has been the use of progressive tension sutures (PTS) and quilting sutures [24, 34–36]. The PTS technique involves the placement of interrupted sutures between the fascia and subcutaneous tissues to reduce the dead space between the planes of tissue created during the dissection [37]. In addition to closing the dead space, it is hypothesized that the tension sutures may also help prevent the shearing effect between the tissue planes in the early healing phase, which may contribute to seroma formation [36, 38]. The use of the PTS technique has not been widely adopted in the US because they add significant time to the procedure and may lead to cosmetic dimpling [24]. The lysine-derived urethane adhesive utilized in the current study accomplishes the same goal of reducing dead space and minimizing shear forces while only taking minimal time to apply at the end of the surgical procedure (mean of 1.2 min).

The mechanism of action of the lysine-derived urethane adhesive utilized in the current study differs from fibrin sealants since the adhesive strongly bonds the tissue planes together and thereby directly reduces the dead space between the elevated tissue flap and the underlying tissue. In contrast, fibrin sealants have been reported to promote the closure of microvascular leaks caused by surgical trauma while also enabling faster healing and revascularization of wound tissue [39, 40]. Few published reports exist that address the efficacy of fibrin sealants in abdominoplasty; however, one study included a set of abdominoplasty patients in its analysis of several procedures where a fibrin sealant was used [41]. The authors reported that there was no reduction in average drain volume or time to drain removal in the abdominoplasty cohort and concluded that the use of fibrin sealants for this procedure is of limited clinical value.

Most previous studies evaluating methods for reducing fluid-related complications have focused on the volume of fluid output from the drains, or on the time to drain removal. Neither of these measures has been shown to have clinical significance in terms of the recovery time of the patient or in their long-term risk of developing an intractable seroma requiring further medical attention. The current study took a different approach by evaluating the number of invasive procedures, which allowed a comparison across groups that directly addresses the frequency of clinical intervention and patient management during the recovery process. This approach also recognizes the fact that having an indwelling drain itself is an invasive procedure, and that drain removal can be perceived by patients to be equally if not more invasive than needle aspiration. This is a highly relevant point, since a seroma cannot be diagnosed in the presence of a drain, and a scoring system must be used that accounts for the presence of a drain to manage post-operative fluid and provides a fair comparison with a needle aspiration. As needle aspiration is a single, low-risk event, we considered a drain removal to be a similar single, low-risk event. A weakness of that measure is that drains present for a longer period time are scored as a single event when removed, despite more days of continuous discomfort for the patient and higher risk of infection. Indeed, a scoring system that accounts for discomfort and inconvenience due to drain maintenance would be useful but more difficult to validate and compare with singular self-limited procedures such as needle aspiration.

The lysine-derived urethane adhesive utilized in our study is biocompatible and has a degradation rate similar to other biologically resorbable polymers. Because the molecule was designed with a lysine core, breakdown products are benign with the majority of the adhesive broken down into lysine derivatives, carbon dioxide, and ethanol, with small amounts of the original sugar-like molecules. Extensive biocompatibility studies, subchronic and chronic toxicity studies (up to 1 year), reproductive toxicity ,and carcinogenicity testing have documented that the adhesive is non-toxic and that it meets all requirements of the International Standard ISO 10993: Biological Evaluation of Medical Devices, which is used by regulatory authorities including the FDA to establish product safety.

Because this clinical study was the first to explore the use of this surgical adhesive without the use of drains, the study population was limited. It is well documented in the literature that post-bariatric patients with massive weight loss are more prone to postsurgical complications, and so these high-risk patients were excluded from the study population. Also, because a variety of liposuction techniques can potentially be used during abdominoplasty, this additional procedure was excluded to insure a more homogenous patient population. Additional studies would be required to evaluate the use of the adhesive in conjunction with liposuction and in higher risk patients.

While the subject population for this study was limited to those undergoing an elective abdominoplasty procedure, the results associated with the use of the lysine-derived urethane adhesive provide evidence that it may also be an effective alternative to help eliminate the dead space between tissue planes and reduce the fluid-related complications in other large flap procedures. Due to clinical characteristics and the similarity of the tissue planes to be approximated, it would be expected that this adhesive would perform analogously in other procedures such as body contouring, mastectomy, TRAM flap breast reconstruction, inguinal lymph node dissection, and latissimus dorsi flap reconstruction. While initial studies associated with the use of this adhesive for several of these procedures have been promising, additional evidence-based studies are needed to document its clinical utility for a number of additional large flap procedures.

## Conclusion

We show in our prospective study that a lysine-derived urethane adhesive is a safe and effective alternative to drains in patients undergoing elective abdominoplasty and can reduce the number of postsurgical invasive procedures required to prevent or manage fluid accumulation-related complications. We believe the use of this adhesive will safely allow patients to avoid the use of drains in large flap procedures, increasing patient satisfaction and reducing the potential for drain-related complications.
